# Abnormal uterine position during pregnancy secondary to abdominal surgery: Two case reports

**DOI:** 10.1097/MD.0000000000030914

**Published:** 2022-10-07

**Authors:** Huanxi Li, Quanfeng Wu, Wei Wei, Xueyan Lin, Xueqin Zhang

**Affiliations:** a Department of Obstetrics, Women and Children’s Hospital, School of Medicine, Xiamen University, Xiamen, Fujian, China.

**Keywords:** cervix position, late pregnancy, middle pregnancy, uterus position

## Abstract

The umbilical cord is the way to exchange gas, supply nutrients, excrete metabolized. Thrombosis of the umbilical cord leads to fetal hypoxia, which jeopardizes fetal health and can cause fetal death. Umbilical vessel thrombosis, which is rarely reported, is difficult to detect prenatally.

**Patient concerns:**

Both individuals included in this study had a history of abdominal operation before pregnancy. The abnormal uterine position demonstrated in the first patient developed secondary to adhesions between the anterior bladder wall and lower segment of the uterus. As the uterus increased in size in proportion with the gestational age, the uterine body continued to enlarge even as the lower uterine segment remained fixed by the adhesions, which resulted in cervical displacement. In comparison, the abnormal uterine position in the second patient was due to a rare case of uterine incarceration developed.

**Diagnosis:**

Both cases were diagnosed as abnormal uterine position during pregnancy secondary to abdominal surgery.

**Interventions:**

The first patient underwent a cesarean section at 33 weeks and 5 days age of gestation for pregnancy complications. The second patient performed a cesarean section at 37 weeks age of gestation.

**Outcomes:**

Due to reasonable treatment, the 2 cases achieved good maternal-infant outcomes.

**Conclusion:**

Abnormal uterine position during pregnancy should be considered seriously, because it can affect the prognosis of the mother and child. When appropriate, a cesarean section is an effective method for terminating such pregnancies. During cesarean section, a longitudinal skin incision may be more beneficial in avoiding secondary injuries. However, the choice of uterine incision should be adjusted for each patient. Care should be given to prevent postpartum hemorrhage.

## 1. Introduction

Abnormal positions of the uterus in middle and late pregnancy is rarely observed in clinical practice.^[1]^ Its prevalence is unknown and it occurs in women of all ages, parities, and gestations. The etiology is not clear and various risk factors were reported such as fibroids, pelvic adhesions, ovarian cysts, anatomical alteration.^[2–4]^ As the pregnancy progresses, physiological changes in the uterus are affected by many other factors, which can result in abnormal positions of the uterus.

## 2. Case report

**Figure 1.**
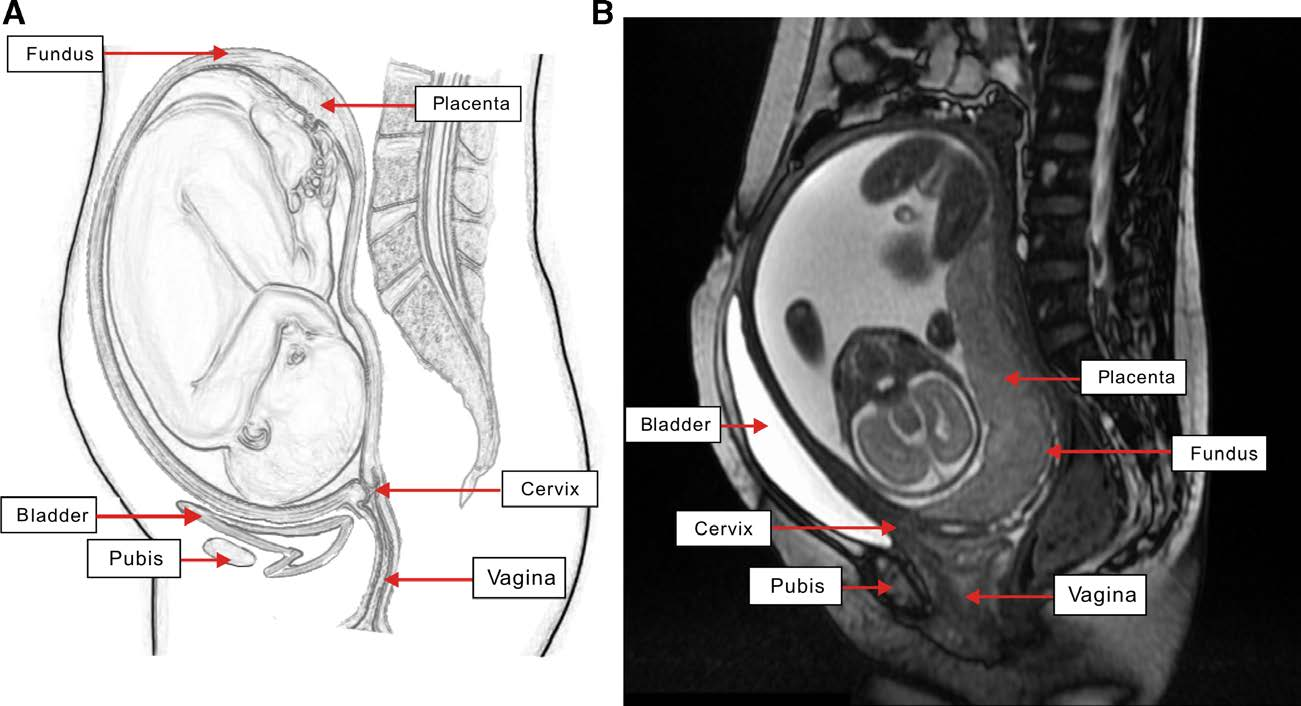
(A) Normal gravid uterus. (B) MRI of the first patient. The image shows the cervical orifice located in front of the anterior inferior wall of the uterus and the cervix and bladder was elongated. MRI = magnetic resonance imaging.

Case 1: The first case was a 40-year-old gravida 3, abortus 0 woman, who presented to the Xiamen Maternity and Child Health Care Hospital for watery vaginal discharge. A review of her past obstetric history revealed that she had undergone a transabdominal ovarian cystectomy 13 years prior. Her current pregnancy was achieved through in vitro fertilization and embryo transfer for tubal factor infertility. The gestational age calculated from her preceding normal menstrual period was 24 weeks and 6 days. She received regular antenatal care at our hospital. The examination on admission demonstrated clear liquid flowing from the vagina, which turned the litmus paper blue. Digital vaginal examination was unable to assess the cervix. A color Doppler ultrasound demonstrated that the placenta was located in the posterior wall, with the lower edge of the placenta completely covering the internal cervix. Magnetic resonance imaging (MRI) revealed that the internal cervical os was located between the posterior wall of the bladder and anterior inferior wall of the uterine body. The internal cervical os was visibly compressed, deformed, and folded against the uterine wall and was positioned higher than the lowest point of the placenta (Fig. 1A and B). The patient was managed conservatively; however, she underwent a cesarean section at 33 weeks and 5 days age of gestation for severe preeclampsia and renal insufficiency. Intraoperatively, the bladder was suspended from the anterior wall of the uterus. A transverse incision was made at the midpoint of the umbilical pubic line to deliver a live baby boy with a birth weight of 2120 g. The appearance, pulse, grimace, activity, and respiration score at 5 minutes was 8. The omentum and intestines were visualized to be closely adherent to the posterior wall of the uterus and cornu uteri, and blood vessels were obviously dilated. We were unable to examine the single remaining ovary and both fallopian tubes. No complications were experienced during the procedure. Intraoperative bleeding was measured at 300 mL. The patient’s postoperative course was smooth, and she was discharged well.

**Figure 2.**
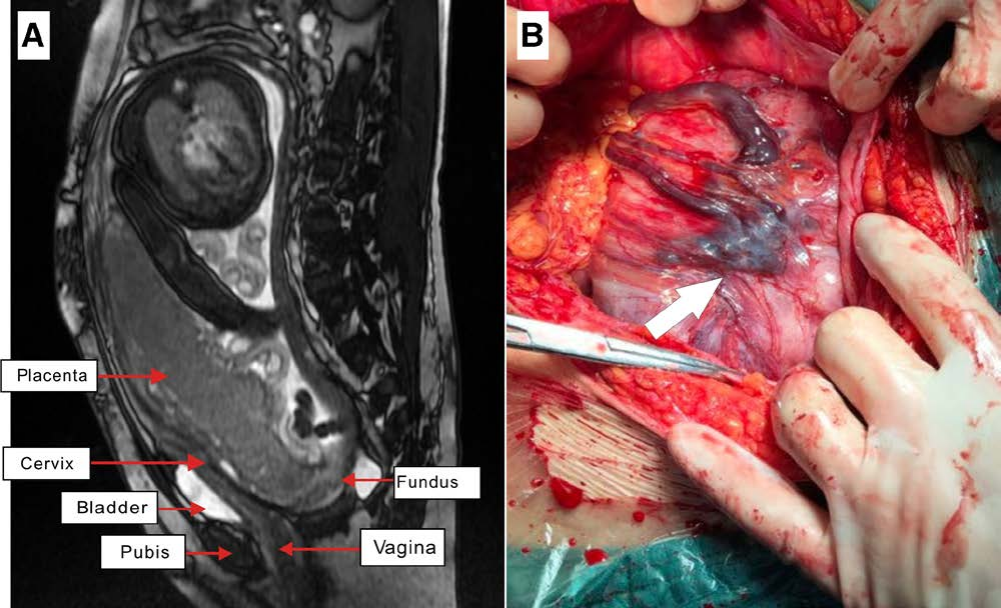
MRI image and intraoperative photograph of the second patient. (A) The image demonstrates extreme retroflexion of the uterus. (B) The photograph shows the upper edge of uterus traversed by a large number of dilated blood vessels. MRI = magnetic resonance imaging.

Case 2: The second case was a 40-year-old primigravid. A review of her obstetric history revealed that the patient had undergone a transabdominal adnexectomy for a giant right ovarian cyst and laparoscopic pelvic adhesiolysis, left ovarian cyst and fallopian tube removal, and uterine fibroid removal 15 and 2 years prior, respectively. Her current pregnancy was achieved through in vitro fertilization and embryo transfer for unexplained primary infertility. At 23 weeks and 4 days age of gestation, routine obstetric ultrasonography demonstrated complete placenta previa and a possible abdominal pregnancy with the fetus located in the abdominopelvic cavity behind the uterus. The size of the uterus was approximately 8.5 cm × 2.4 cm × 5.0 cm, and an endometrial line was visible. A confirmatory MRI was performed, which demonstrated a retroflexed uterus with an intrauterine pregnancy, and complete placenta previa (Fig. 2A). Regular follow-up ultrasonography examinations revealed extreme retroflexion of the uterus. The patient was admitted to the hospital at 36 weeks and 2 days age of gestation. A repeat MRI performed on admission demonstrated the same results. The pregnancy was carried to term well, and a cesarean section was performed at 37 weeks age of gestation. A longitudinal incision was made on the abdomen to expose the uterus. Due to the abnormal position of the uterus, we were unable to identify the normal anatomic landmarks for surgery. We elected to follow the course of the skin incision and incised along the left side of the umbilicus at a length of approximately 5 cm. This clearly exposed the torsion edge of the uterus, which was traversed by a large number of dilated blood vessels (Fig. 2B). We sharply dissected the peritoneum from the uterus and bladder, but were unable to palpate the cervical tissues. Preoperative MRI demonstrated that the fetus was in a transverse position with the head on the left side. We incised the uterine wall at the expected fetal head position and delivered a baby boy with a 5 min appearance, pulse, grimace, activity, and respiration score of 10 and birth weight of 2910 g. Intraoperatively, our incision was located in the lower uterine segment. The inner cervical canal was visible approximately 1 cm below the lower edge of the uterine incision, and the cervical canal was approximately 10 cm long. The uterus was severely retroflexed, so that adhesions were noted between the posterior uterine and posterior vaginal walls. The uterus also showed adhesions with the left adnexa, omentum, intestines, and pelvic wall, which resulted in very poor mobility. The placenta was noted along the left, posterior wall of the uterus, and its lower edge was approximately 1 cm away from the inner cervical canal. The procedure was uncomplicated. Intraoperative bleeding was measured at 400 mL. The postoperative course was smooth, and the patient was discharged well.

## 3. Discussion

### 3.1. Causes of abnormal uterine position during pregnancy

During pregnancy, the enlarged uterus rises from the pelvic cavity to occupy a part of the abdomen. The majority of this growth occurs in the uterine isthmus, which stretches and becomes thinner. The uterus also expands downwards into the uterine cavity and stretches 7 to 10 cm during labor to become a part of the birth canal. During pregnancy, the uterus also demonstrates slight dextrorotation, because the sigmoid colon and rectum on the left side of the pelvis limits uterine growth in that direction. As the pregnancy progresses, physiological changes in the uterus are affected by many other factors, which can result in abnormal positions of the uterus.^[5]^ The abnormal uterine position demonstrated in the first patient developed secondary to adhesions between the anterior bladder wall and lower segment of the uterus. As the uterus increased in size in proportion with the gestational age, the uterine body continued to enlarge even as the lower uterine segment remained fixed by the adhesions, which resulted in cervical displacement. In comparison, the abnormal uterine position in the second patient was due to a rare case of uterine incarceration. In a typical pregnancy with a retroverted or retroflexed fundi, as the uterus enlarges from the hollow of the sacrum, it assumes an anterior ventral position, which spontaneously corrects the uterine malposition. However, in rare cases, the long axis of the uterus cannot extend into the abdominal cavity, so that the uterus grows dorsally or ventrally instead. This causes the uterus to be confined within the uterine rectal or bladder lacunae, resulting in an incarcerated gravid uterus. As the gestational age increases, the fundus becomes wedged below the sacral promontory, where it can continue to enlarge for a period of time,^[6]^ and the cervix shifts to the same side or above of the pubic symphysis. Eventually, the posterior pelvic space becomes inadequate, and the anterior lower uterine wall thins and sacculates into the upper abdomen to accommodate the products of conception. Concomitantly, both the bladder and cervix are pulled into the abdominal cavity toward the umbilicus. The cervix can stretch to 10 cm or more in length, such that the internal os can be located above the pubic symphysis and, occasionally, the bladder.^[7]^ Risk factors for an incarcerated gravid uterus include previous pelvic surgery, pelvic inflammatory disease, adhesions from endometriosis, large uterine fibroids, and uterine malformations, among others.^[8]^ An incarcerated gravid uterus can also occur without predisposing factors, but this is not common.^[9]^ We believe that the incarcerated gravid uterus in the second patient developed secondary to her history of multiple pelvic and abdominal surgeries.

### 3.2. Clinical manifestations of abnormal uterine position during pregnancy, and auxiliary examinations

Most patients with abnormal cervical positions due to pelvic adhesions have no obvious clinical manifestations during pregnancy. Abnormal positions of the cervix are most often diagnosed by incidental ultrasound examination or when the cervix cannot be assessed during digital vaginal examination. In cases of an incarcerated gravid uterus, patients may present with urinary retention and hydronephrosis, because the cervix and upper vagina are displaced superiorly, which lifts the bladder neck and compresses the urethra.^[10]^ Overall, ultrasound is not sensitive for abnormal uterine positions and often misdiagnoses the condition. However, ultrasound may detect abnormally positioned ovarian blood vessels or changes in the placental position, which could indicate an underlying pathology of abnormal uterine position. In comparison, MRI demonstrates good soft tissue resolution and can accurately determine the anatomical structure and hierarchy of the various organs in the pelvis.

### 3.3. Clinical treatment strategy

Any abnormalities in the position or morphology of the uterus during pregnancy should arouse great suspicion and an active search for the underlying cause, because the underlying cause will determine the definitive treatment. In most cases of abnormal cervical position caused by pelvic adhesions, no special treatment is usually required during pregnancy. However, in more complicated cases, the termination of the pregnancy may be required and should be planned according to obstetric indications. According to some reports, an incarcerated gravid uterus should be terminated at 36 weeks age of gestation.^[11]^

A cesarean section is the recommended mode of delivery for pregnancies with abnormal uterine position or morphology. During the preoperative period, the clinical physician should familiarize himself with the atypical location of the uterus and placenta and prepare blood as a precaution for a more complex intraoperative or postoperative course. The physician should also make sure to prepare the patient and her family for possible complications that can arise during surgery. A vertical hypogastric incision is the most ideal, because it provides a good surgical field for separating adhesions while avoiding organ damage. However, the chosen incision should be based on the intraoperative situation, position of the bladder, and MRI results. The location of the incision is usually determined by the relative position of the bladder.^[12]^ Abnormal uterine and cervical positions that developed due to the adhesions in the pelvic cavity are also at high risk for postpartum hemorrhage. Postpartum hemorrhage should be actively prevented during surgery by ligating the uterine artery, suturing the uterine strap, or even performing hysterectomy. We initially had difficulty with the first patient because of the lack of surgical landmarks. We determined the position of the bladder by filling it with water to avoid bladder damage. In the second case, the incarcerated gravid uterus pulled the cervix up into the abdominal cavity and folded the uterine body behind the cervix. If we entered the abdomen according to the routine method, it would have easily caused cervical injury and prevented the delivery of the fetus. Therefore, we elected to create our incision near the umbilicus based on the preoperative MRI scans and extended this incision to the lower uterine segment to deliver the fetus efficiently.

## 4. Conclusion

Based on these two cases, abnormal uterine positions during pregnancy should be considered seriously, and the underlying cause should be actively searched for at the earliest. When necessary, cesarean section is an appropriate method for terminating these pregnancies. During cesarean section, a longitudinal skin incision is superior for avoiding possible secondary injuries; however, the chosen incision should always be based on the individual presentation of the patient. Meticulous care should be given in preventing postpartum hemorrhage.

## Acknowledgements

This work was supported by Women and Children’s Hospital, School of Medicine, Xiamen University.

## Authors’ contributions

LHX performed the surgery, and coordinate the patient management. WQF analyzed and interpreted the patient data regarding the disease and drafted the manuscript. WW and LXY contributed substantially in writing the manuscript. ZXQ designed the work, coordinate the data interpretation, confirmed the accuracy and originality of the data, and drafted and substantially revised the manuscript. All authors reviewed draft versions of the manuscript and approved the final version.

**Conceptualization:** Huanxi Li, Xueqin Zhang.

**Data curation:** Huanxi Li.

**Formal analysis:** Wei Wei.

**Investigation:** Wei Wei.

**Methodology:** Wei Wei.

**Project administration:** Wei Wei.

**Resources:** Xueyan Lin.

**Software:** Xueyan Lin.

**Supervision:** Xueyan Lin.

**Validation:** Xueyan Lin.

**Visualization:** Quanfeng Wu, Xueyan Lin.

**Writing – original draft:** Quanfeng Wu.

**Writing – review & editing:** Huanxi Li, Xueqin Zhang.
